# Supplementary material for: The impact of land use on non-native species incidence and number in local assemblages worldwide
Source: Nat Commun. 2023 Apr 12;14:2090. doi: 10.1038/s41467-023-37571-0 (PMC10097616; doi:10.1038/s41467-023-37571-0)
Supplement: Supplementary file 1 — Supplementary Information [file 41467_2023_37571_MOESM1_ESM.pdf]

Supplementary Information file

The impact of land use on non-native species incidence and number in local assemblages worldwide

Daijun Liu[1*], Philipp Semenchuk[1,2], Franz Essl[1], Bernd Lenzner[1], Dietmar Moser[1], Tim M. Blackburn[3,4], Phillip Cassey[5], Dino Biancolini[6,27], César Capinha[7,8], Wayne Dawson[9], Ellie E Dyer[10,11], Benoit Guénard[12], Evan P. Economo[13,14], Holger Kreft[15,16], Jan Pergl[17], Petr Pyšek[17,18], Mark van Kleunen[19,20], Wolfgang Nentwig[21], Carlo Rondinini[6], Hanno Seebens[22], Patrick Weigelt[15,16,23], Marten Winter[24], Andy Purvis[25,26], Stefan Dullinger[1]

Supplementary Figures

Figure caption

Supplementary Figure 1. Geographical distribution of local assemblages with at least one non-native species for each taxon separately.

Supplementary Figure 2. Odds ratio of non-native incidence in local assemblages in response to changes in LU-type and LU-intensity separately.

Supplementary Figure 3. Number of non-native species for assemblages with at least one non-native species in response to LU-type and LU-intensity separately.

Supplementary Figure 4. Non-native proportions of local assemblages in response to changes in LU-type and LU-intensity separately.

Supplementary Figure 5. Spatial cross-validation of the full model results with respect to spatial biases from uneven sampling across biomes.

Supplementary Figure 6. Spatial cross-validation of models with respect to spatial biases from uneven sampling across biomes using LU-type as the only fixed effect.

Supplementary Figure 7. Spatial cross-validation of model results with respect to the location of the assemblage on an island or on the mainland.

Supplementary Figure 8. Spatial cross-validation of models with respect to the location of the assemblage on an island or on the mainland using LU-type as the only fixed effect.

Supplementary Tables

Table title

Supplementary Table 1. The classification of LU-types and – intensity levels in our analysis.

Supplementary Table 2. Sources of regional non-native species distribution for the taxa analysed

Supplementary Table 3. Invalid or unresolved species in PREDCITS assemblages that were removed from analyses

Supplementary Table 4. Percentages of local assemblages with non-natives across all taxa and for each taxon separately.

Supplementary Table 5. Odds ratios of non-native incidence in local assemblages in response to the interaction of LU-type and LU-intensity.

Supplementary Table 6. Odds ratios of non-nativeincidence in local assemblages in response to LU-type.

Supplementary Table 7. Comparison of models of non-native incidence, number and proportion that do or do not include the assignment of assemblage locations to one of the 14 biomes distinguished in Supplementary References[13] into the random effects structure.

Supplementary Table 8. Cross-validation of robustness of coefficient estimates in the models of non-native incidence, number and proportion computed by excluding assemblages from one biome in turn.

Supplementary Table 9. Comparison of model results for non-native incidence, number and proportions without and with including the size of the sampling area of assemblages as additional fixed-effects predictor

Supplementary Table 10. Comparison of models of non-native incidence, number and proportion that either account or do not account for the location of the assemblage on an island or on the mainland

Supplementary Table 11. Test of robustness of coefficient estimates in the models of non-native incidence, number and proportion computed by excluding assemblages from t islands.

Supplementary Table 12. Number of non-native species in local assemblages in response to the interaction of LU-type and LU-intensity.

Supplementary Table 13. Number of non-native species in local assemblages in response to LU-type.

Supplementary Table 14. Proportion of non-native species in local assemblages in response to the interaction of LU-type and LU-intensity.

Supplementary Table 15. Proportion of non-native species among all species in local assemblages in response to LU-type.

Supplementary Table 16. Number of all assemblages and of those with at least one non-native species, given separately for the LU-types.

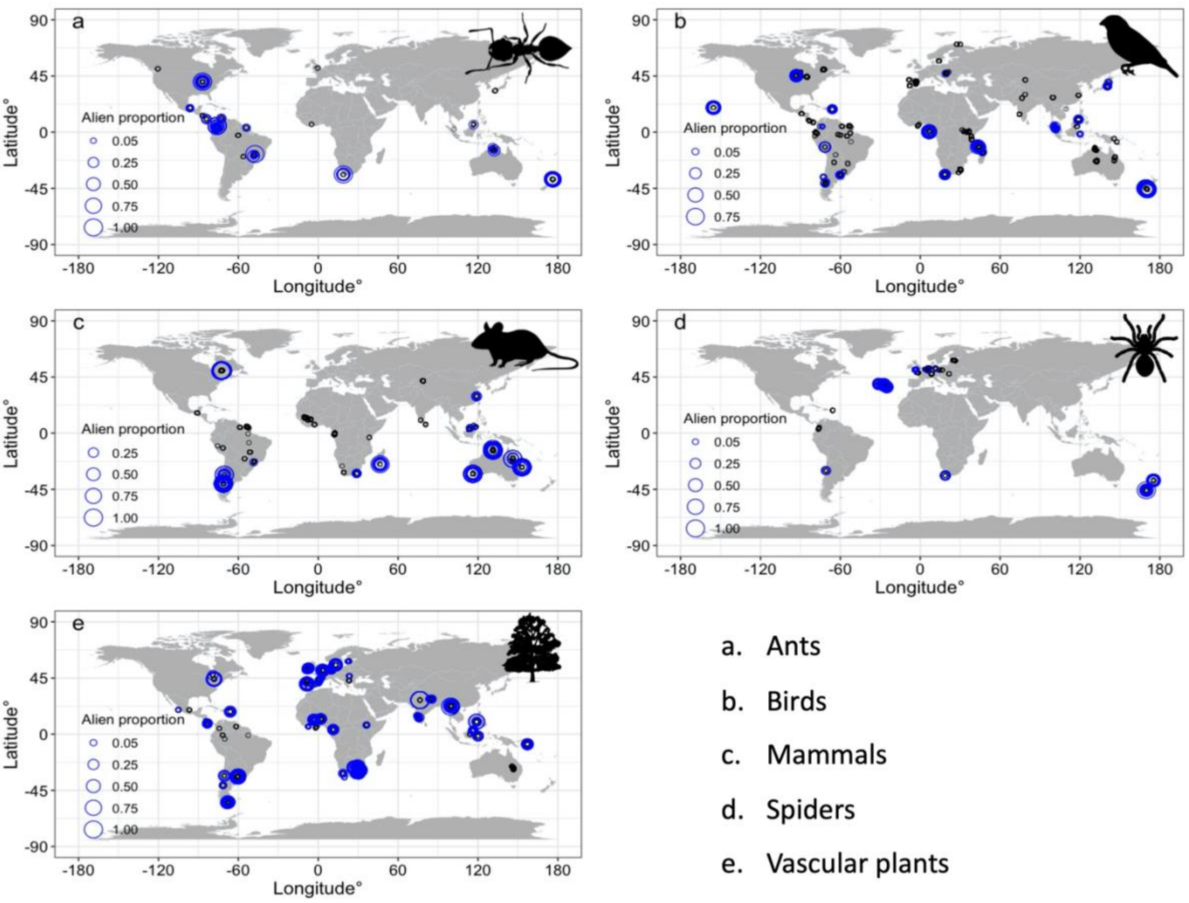

Supplementary Figure 1. Geographical distribution of local assemblages with at least one non-native species for the taxon. A, ants; b, birds; c, mammals; d; spider; e, vascular plants. The colors indicate distribution of assemblages with at least one non-native species (blue points) and the assemblage with no non-native species (black points); respectively. Silhouette illustrations for the taxa are from PhyloPic (http://phylopic.org), contributed by various authors under public domain license.

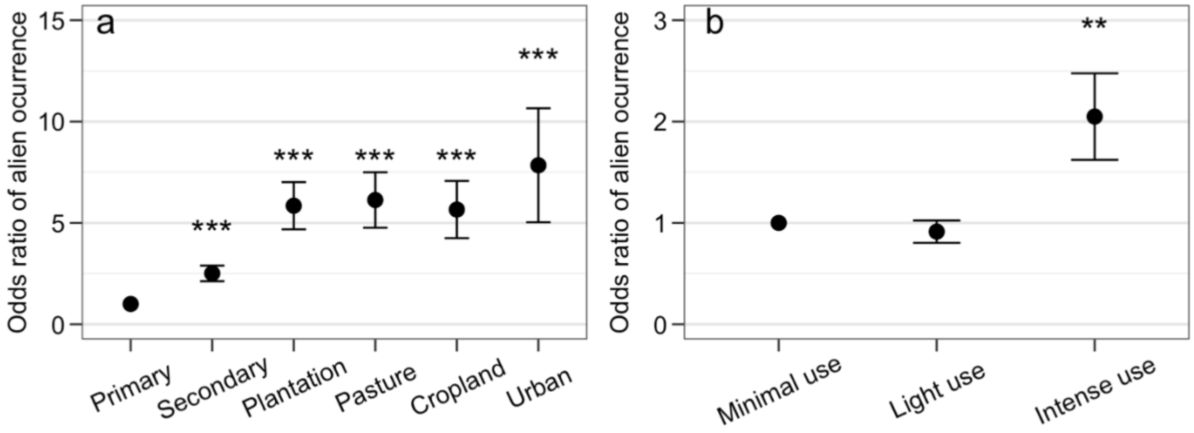

Supplementary Figure 2. Odds ratio of non-native incidence in local assemblages in response to LU-type (a) and LU-intensity (b) separately. The logistic generalized linear mixed effects model with LU-type (a) and with LU-intensity (b) were used separately (n=11,713, 9869; respectively). Odds ratios (the means and standard errors) were back-transformed pairwise contrasts to the reference level ‘primary vegetation’ and ‘minimal use’; respectively. The asterisks in the figure indicate significant differences (p values: **<0.01 and ***<0.001; respectively).

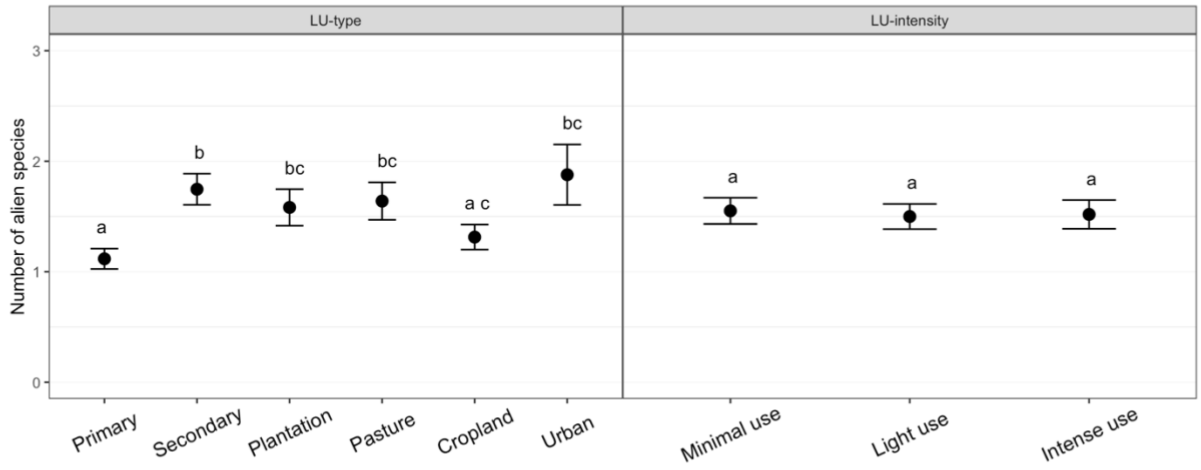

Supplementary Figure 3. Number of non-native species for assemblages with at least one non-native species in response to LU-type (left panel) and LU-intensity (right panel) separately. Numbers of non-native species were back-transformed from a generalized linear mixed effects model (GLMM) with a compact letter display of all pairwise comparisons of estimated marginal means (n=2450, 2314; respectively). The means denoted by a different letter indicate significant differences ($p<0.05$). Data are presented as mean values and standard errors.

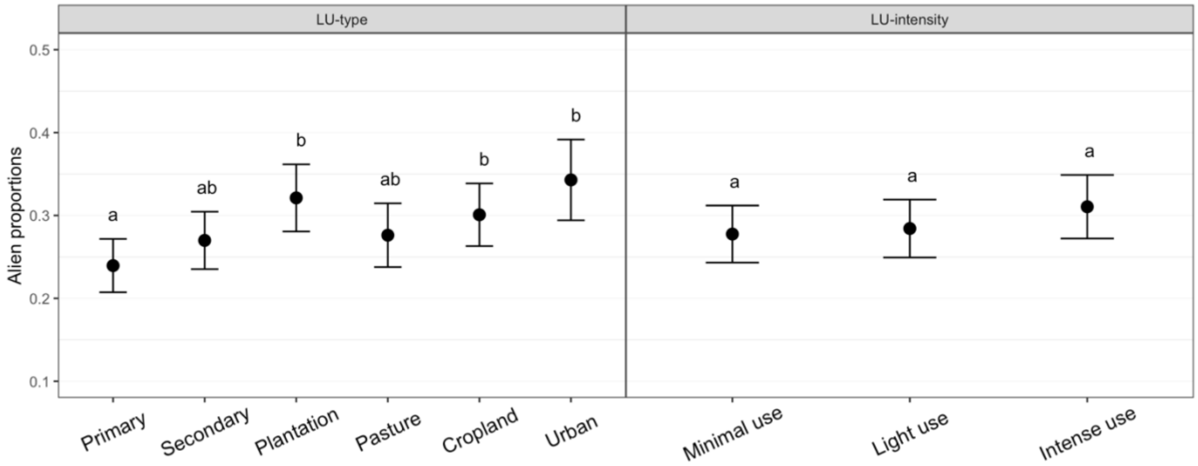

Supplementary Figure 4. Non-native proportions of local assemblages in response to changes in LU-type (left panel) and LU-intensity (right panel) separately. Non-native proportions were back-transformed from a generalized linear mixed effects model (GLMM) and a compact letter display of all pairwise comparisons of estimated marginal means (n=2,450, 2,314; respectively). Means denoted by a different letter are significantly different ($p<0.05$). Data are presented as mean values and standard errors.

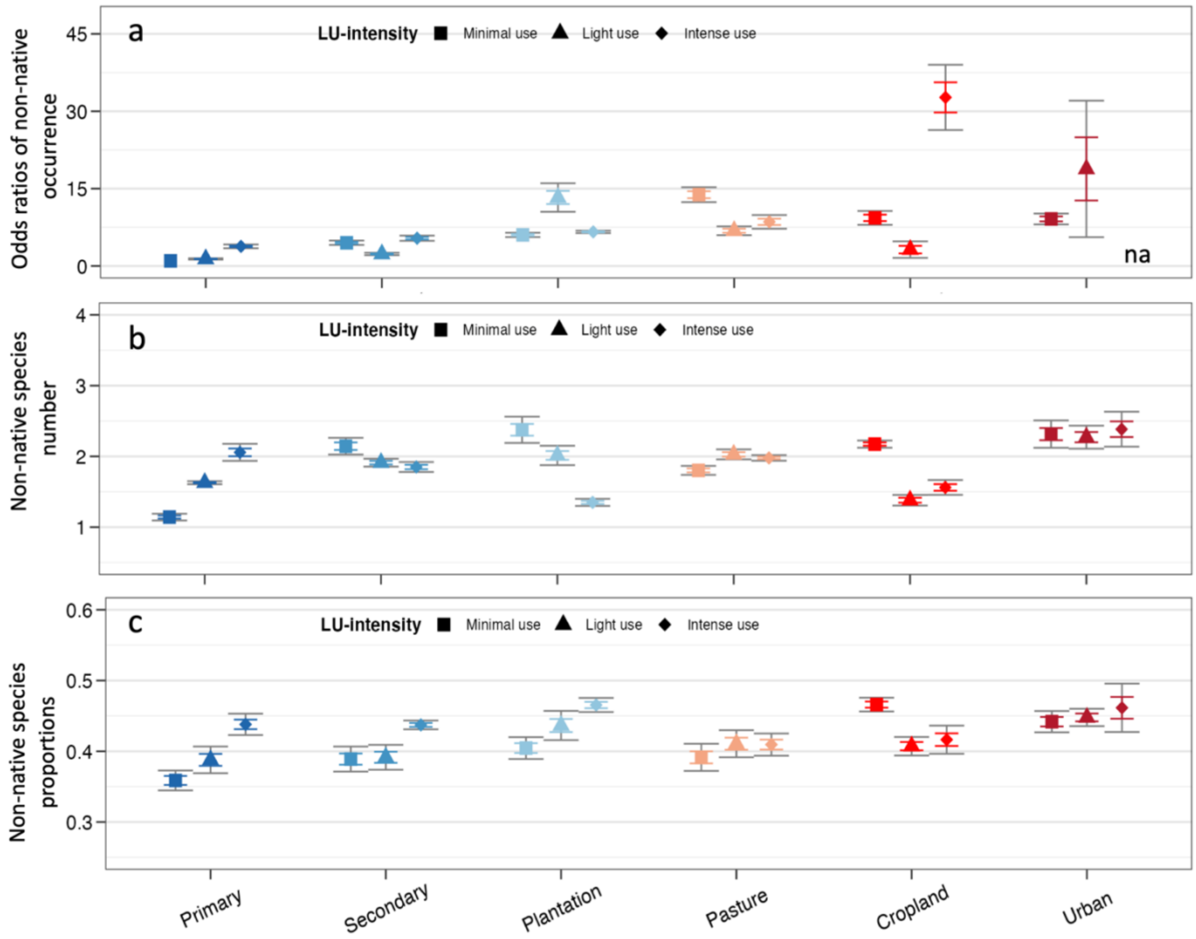

Supplementary Figure 5. Spatial cross-validation of the full model results with respect to spatial biases from uneven sampling across biomes. Models were re-run by excluding all assemblages from one biome in turn. a, models of non-native incidence (n=11,693); b, non-native species number (n=2,314) and c, non-native species proportions in local assemblages (n=2,314). Symbols show the mean coefficient values across all models, grey bars their 95% confidence intervals and coloured bars the mean standard errors. All models used LU-type, LU-intensity and their interaction as fixed effects and study-site blocks nested in study sites (SSB/SS) as random factors. Models of non-native incidence were run 14 times, corresponding to the 14 biomes in Supplementary References[13]. Models of non-native species number and proportions were run 11 times because some biomes had too low numbers of assemblages. Coefficient estimates of statistical models are back-transformed pairwise contrasts to the reference level ‘primary vegetation under Minimal use’ in each model. The ‘na’ in (a) indicates that urban assemblages under intense use could not be included due to lack of variation in the response (all assemblages had alien species).

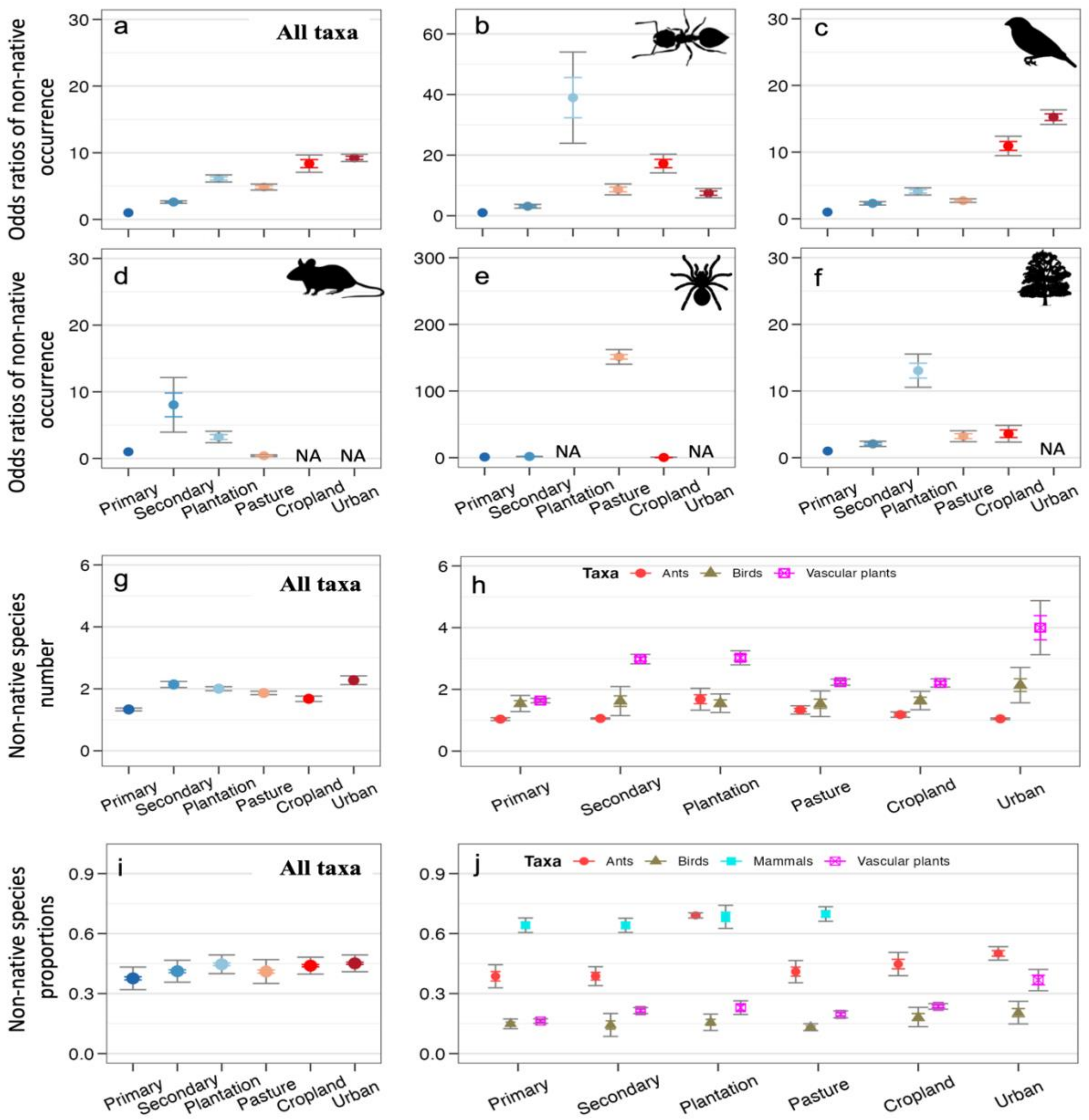

Supplementary Figure 6. Spatial cross-validation of models with respect to spatial biases from uneven sampling across biomes using LU-type as the only fixed effect. a-f, models of non-native incidence across all taxa (n=11,713) and each taxon separately (n=407, 3,978, 1,114, 762 and 4,453; respectively); g,h, models of non-native species number and i, j models of non-native species proportions across all taxa (n=2,450) and for each taxon separately (n=113, 457, 292, 182 and 1,406; respectively). Symbols show the mean coefficient values across all models, grey bars their 95% confidence intervals and coloured bars the mean standard errors. The values are back-transformed pairwise contrasts to the reference level 'primary vegetation'.The NA in (d, e, f) indicates that these LU-types were removed from the analysis due to low numbers of the assemblages (see Methods for details). In case of non-native species number, mammals were not analyzed due to low variance in species number per assemblage. Spiders were not analyzed due to low sample size. Silhouette illustrations for the taxa are from PhyloPic (http://phylopic.org), contributed by various authors under public domain license.

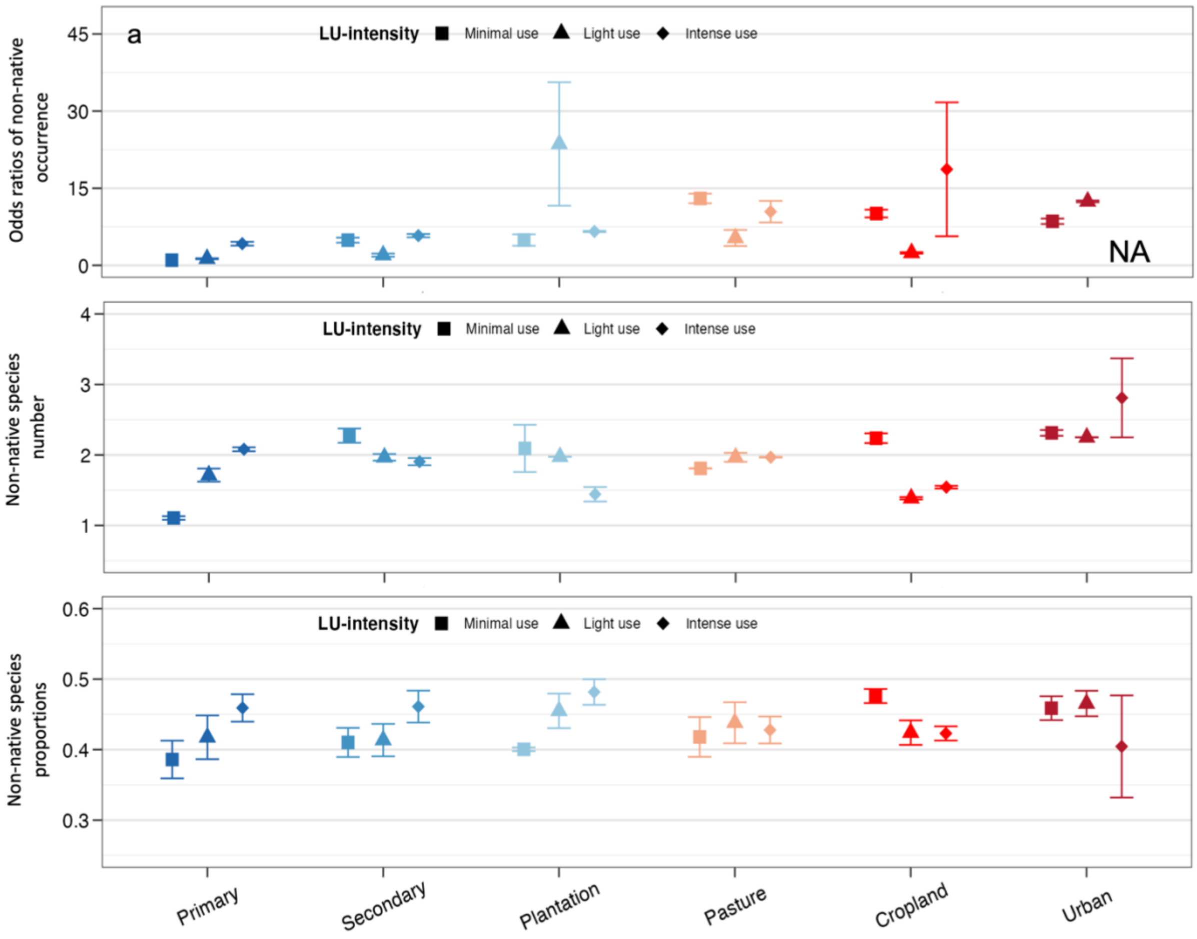

Supplementary Figure 7. Spatial cross-validation of model results with respect to the location of the assemblage on an island or on the mainland. We re-run two full models, one including all assemblages and the other one all but those from islands. a, models of non-native incidence (n=11,693); b, non-native species number (n=2,314) and c, non-native species proportions (n=2,314). Symbols show the mean coefficient values across the two models, coloured bars indicate the mean standard errors. All models used LU-type, LU-intensity and their interaction as fixed effects and study-site blocks nested in study sites (SSB/SS) as random factors. Coefficient estimates of statistical models are back-transformed pairwise contrasts to the reference level 'primary vegetation under Minimal use' in each model. The 'NA' in (a) indicates that urban assemblages under intense use could not be included due to lack of variation in the response (all assemblages had alien species).

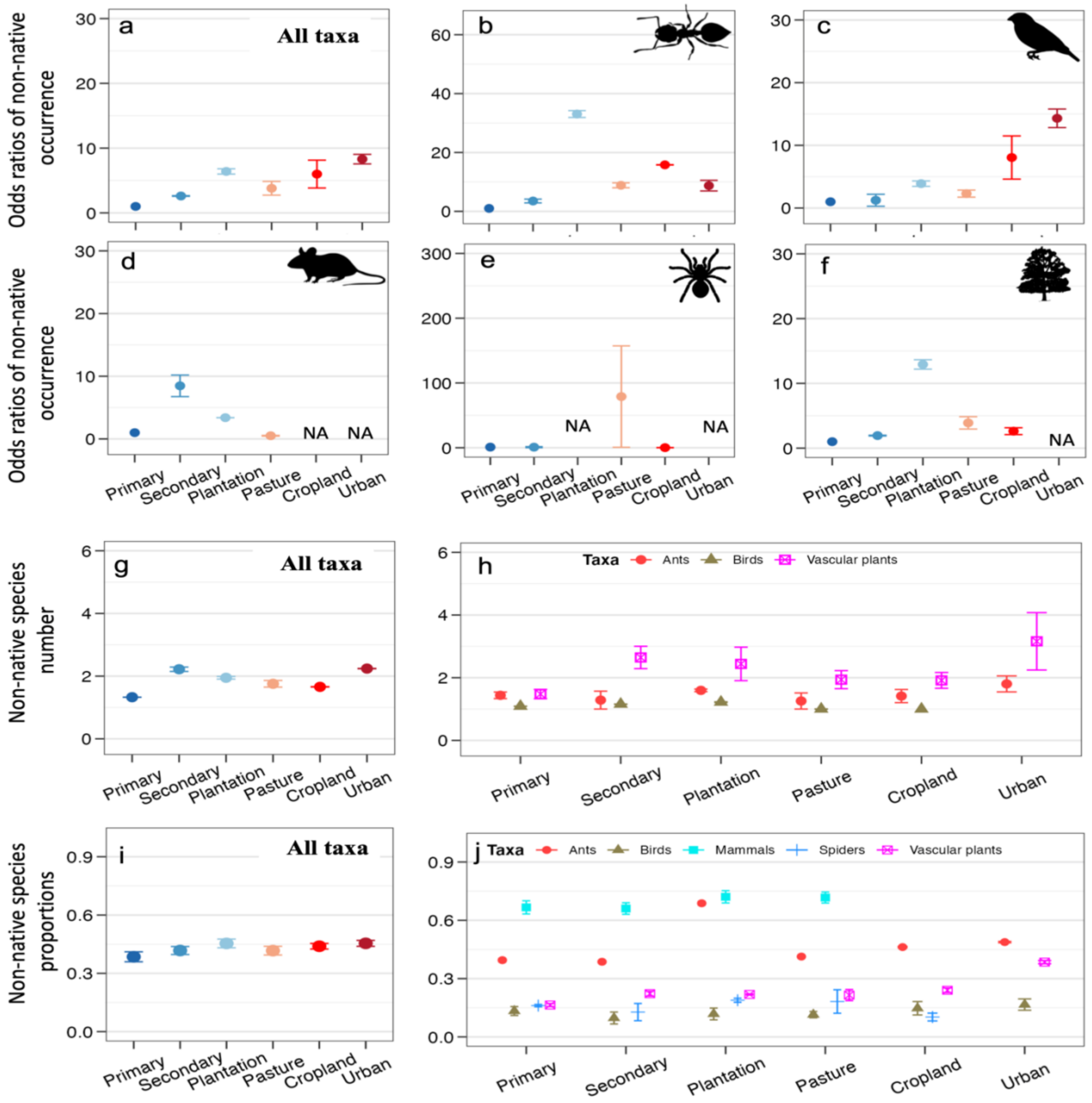

Supplementary Figure 8. Spatial cross-validation of models with respect to the location of the assemblage on an island or on the mainland using LU-type as the only fixed effect. We re-run two full models, one including all assemblages, and one all but those from islands. a-f, models of non-native incidence across all taxa (n=11,713) and each taxon separately (n=407, 3,978, 1,114, 762 and 4,453; respectively); g,h, models of non-native species number and i, j models of non-native species proportions across all taxa (n=2,450) and for each taxon separately (n=113, 457, 292, 182 and 1,406; respectively). Symbols show the mean coefficient values across the two models, coloured bars indicate the mean standard errors. The values are back-transformed pairwise contrasts to the reference level ‘primary vegetation’.The ‘NA’ in (d, e, f) indicates that these LU-types were removed from the analysis due to low numbers of the assemblages (see Methods for details). In case of non-native species number, mammals were not analyzed due to low variance in species numbers per assemblage. Spiders were not analyzed due to the model convergence by excluding the assemblages from island. Silhouette illustrations for the taxa are from PhyloPic (http://phylopic.org), contributed by various authors under public domain license.

Supplementary Table 1. The classification of LU-types and -intensity levels as used in our analysis. Three stages of secondary successional (young/intermediate/mature) were combined, six LU-types and three LU-intensity levels. The description of LU-type and -intensity levels follows Supplementary References[1,2].

Land use	Land use types in the PREDICTS dataset	Minimal use	Light use	Intense use
**Primary**	**Primary vegetation**: natural habitats not known to have ever been completely destroyed by human actions or natural events that are not part of the natural disturbance regime of the respective ecosystems. Includes synonyms such as "ancient woodlands", "old-growth forests" or "natural grasslands"	Any disturbances identified are minor.	One or more disturbances of moderate intensity.	One or more disturbances that are severe enough to markedly change the natural ecosystems. Primary sites in urban areas are intensely used.
**Secondary**	The original primary vegetation was completely destroyed. **Young secondary vegetation**: a simple architecture representing an early successional stage; forest stands less than 10 years old in the tropics or 30 years old in temperate regions. **Intermediate secondary vegetation**: a mixed architecture showing a mid-successional stage, roughly corresponding to stands aged 10-30 years in the tropics or 30-75 years in temperate regions. **Mature secondary vegetation**: structure approaching to primary vegetation, which is a completed succession; such forest stands would typically be at least 30 years old in the tropics or 75 years old in temperate regions.	Same as above	Same as above	Same as above
**Plantation**	**Plantation forest:** previously cleared areas that people have planted with crop trees or crop shrubs for commercial or subsistence harvesting of wood and/or fruit, including e.g. oil palm, coffee, fruits and	Extensively managed or mixed timber, fruit/coffee, oil-palm or rubber plantations in natural understorey,	Monoculture fruit/coffee/ rubber plantations with limited pesticide input, or mixed species	Monoculture fruit/coffee/rubber plantations with significant pesticide input. Monoculture timber with

	timber plantations.. The species planted may or may not be native.	which are not treated with pesticide fertilizer, and which have not been clear-felled (< 20 years).	plantations with significant inputs, or monoculture timber and oil-palm plantations have not been clear-felling (<20 years).	similarly aged trees or timber/oil-palm plantations with extensive recent clear-felling (<20 years).
**Pasture**	**Pasture:** land where livestock is known to be grazed regularly or permanently (i.e. grazing grasslands). The plant species may be predominantly native (as in rangelands) or strongly associated with humans (as in European-style pastures).	With minimal input of fertilizer and pesticide, and with low stock intensity.	With significant input of fertilizer or pesticide, or with high stock density.	With significant input of fertilizer or pesticide, and high stock density that may cause significant disturbance or stop regeneration of vegetation.
**Cropland**	**Cropland:** land that has planted with herbaceous crops (i.e. agricultural land), even if these crops will be fed to livestock once harvested	Low-intensity farms, typically with small fields, mixed crops, little or no pesticide, or no inorganic fertiliser use.	Medium intensity farming, typically with large fields, annual ploughing, little or no pesticide, or no inorganic fertiliser use.	High-intensity monoculture farming, typically showing the features of large fields, annual ploughing, pesticide application, inorganic fertiliser use and no crop rotation.
**Urban**	**Urban**: human habitation and/or buildings, where the primary vegetation has been removed.	Extensive managed green space and villages.	Suburban, garden or small managed or unmanaged green space in cities.	Fully Urban with no significant green spaces.

Supplementary Table 2. Sources of regional non-native species distribution for the taxa analysed. Ecoregions mentioned in the description refer to the Biodiversity Information standards (TDWG) level 4.

Taxa	Database	Descriptions	References
1.Ants	Global Ant Biodiversity Informatics (GABI) project database	Compilation of 4822 occurrences of 303 non-native ant species across 455 ecoregions.	Supplementary Reference[3]
2.Birds	Global Alien Invasions Atlas (GAVIA)	Global dataset of 27,723 distribution records for 971 non-native bird species. However, non-native birds that have no range map exist, which are listed in a table with information on where they occur as non-natives in text form, with very different geographical precision. These species are not included. At last, 2691 occurrences of 361 non-native bird species across 517 ecoregions are included in our analysis. These occurrences and species are consistent with the data in the study of Dawson *et al.*, 2017.	Supplementary Reference[4]
3.Mammals	DAMA: the global Distribution of Alien Mammals database + nine ubiquitous non-native mammals: *Bos taurus*, *Canis familiaris*, *Capra hircus*, *Equus asinus*, *Equus caballus*, *Felis catus*, *Mus musculus*, *Ovis aries*, *Sus scrofa.*	Contains comprises 2726 range polygons for 239 established non-native mammal species, covering 199 Countries, 2190 level 1 administrative areas and 11 zoogeographic realms.	Supplementary References[5-9]
4.Spiders	Global spider	Compilation of 2,139 records of 207 non-native spider species across 353 ecoregions.	Supplementary Reference[10]
5.Vascular plants	Global Naturalized Alien Flora (GloNAF) Database	Contains 270,168 occurrence records of 15,111 non-native vascular plant species in c. 448 geopolitical regions.	Supplementary Reference[11,12]

Supplementary Table 3. Invalid or unresolved species in PREDCITS assemblages that were removed from analyses..

Species number	Taxonomic group	**Species name in PREDICTS**
1	Ants	Hyform 0
2	Ants	Solenopsis dip
3	Ants	Lasius lasius
4	Ants	Myrmica myrmica
5	Ants	Winged Ants
6	Ants	Hypoponera media
7	Ants	Hypoponera negra
8	Ants	Linepithema media
9	Ants	Octostruma roja
10	Ants	Paratrechina coxas
11	Ants	Pheidole espinigruesa
12	Ants	Pheidole media
13	Ants	Pheidole olorosa
14	Ants	Pheidole pequena
15	Ants	Rogeria negra
16	Ants	Solenopsis abdomen
17	Ants	Solenopsis amarilla
18	Ants	Crematogaster brillante
19	Ants	Crematogaster espina
20	Ants	Gnamtogenys grande
21	Ants	Gnamtogenys peciolo
22	Ants	Strumigenys media

23	Ants	Pheidole diminuta
24	Ants	Strumigenys amarilla
25	Ants	Dolichoderus nahe
26	Ants	Queen 295
27	Ants	Queen 296
28	Ants	Queen 297
29	Ants	Camponotus forelophilus
30	Ants	Pyramica wheeleri
31	Birds	Canary blue
32	Birds	Common stone
33	Birds	Eurasian tree
34	Birds	Imperial pigeon
35	Birds	Indian tree
36	Birds	Large yellow
37	Birds	Red headed
38	Spiders	Cicurina collaris
39	Spiders	Cicurina fossor
40	Spiders	Arsalt 0
41	Spiders	Nephila like
42	Spiders	Anasaetis tristrieata
43	Spiders	Ocyrocera aurora
44	Spiders	Pseudosparianthes jayayuae
45	Spiders	Siloca minimus
46	Spiders	Pterotrichia varia
47	Spiders	Anyphaenidae indet

48	Spiders	Cyrtaucheniidae indet
49	Spiders	Dipluridae indet
50	Spiders	Gamasomorphinae indet
51	Spiders	Idiopidae indet
52	Spiders	Linyphiidae indet
53	Spiders	Lycosidae indet
54	Spiders	Ochyroceratidae indet
55	Spiders	Oonopinae indet
56	Spiders	Salticidae indet
57	Spiders	Theraphosidae indet
58	Spiders	Theridiosomatidae indet
59	Vascular plants	Ge sp
60	Vascular plants	Naked tree
61	Vascular plants	U u
62	Vascular plants	Eugenia conformis
63	Vascular plants	Pouteria edelcana
64	Vascular plants	Acacia unknown
65	Vascular plants	Aniba squadensis
66	Vascular plants	Acacia loofblaarplant
67	Vascular plants	Aster lobelia
68	Vascular plants	Morella gladdeblaar
69	Vascular plants	Cf fabaceae
70	Vascular plants	Cyperaceae family
71	Vascular plants	Restionaceae family
72	Vascular plants	Acanthaceaegenus sp1

73	Vascular plants	Acanthaceaegenus sp2
74	Vascular plants	Acanthaceaegenus sp3
75	Vascular plants	Araceaegenus sp01
76	Vascular plants	Araceaegenus sp02
77	Vascular plants	Araceaegenus sp03
78	Vascular plants	Araceaegenus sp04
79	Vascular plants	Araceaegenus sp05
80	Vascular plants	Araceaegenus sp06
81	Vascular plants	Araceaegenus sp07
82	Vascular plants	Araceaegenus sp08
83	Vascular plants	Araceaegenus sp09
84	Vascular plants	Araceaegenus sp10
85	Vascular plants	Asteraceaegenus sp1
86	Vascular plants	Asteraceaegenus sp2
87	Vascular plants	Asteraceaegenus sp3
88	Vascular plants	Asteraceaegenus sp4
89	Vascular plants	Asteraceaegenus sp5
90	Vascular plants	Euphorbiaceaecfgenus sp1
91	Vascular plants	Euphorbiaceaecfgenus sp2
92	Vascular plants	Gesneriaceaegenus sp1
93	Vascular plants	Gesneriaceaegenus sp2
94	Vascular plants	Lamiaceaegenus sp1
95	Vascular plants	Lamiaceaegenus sp2
96	Vascular plants	Lamiaceaegenus sp3
97	Vascular plants	Liliaceae cultivated

98	Vascular plants	Malphigiaceaegenus sp1
99	Vascular plants	Malphigiaceaegenus sp2
100	Vascular plants	Malphigiaceaegenus sp3
101	Vascular plants	Malvaceaegenus sp2
102	Vascular plants	Malvaceaegenus sp3
103	Vascular plants	Melastomataceaegenus sp1
104	Vascular plants	Melastomataceaegenus sp2
105	Vascular plants	Melastomataceaegenus sp3
106	Vascular plants	Melastomataceaegenus sp4
107	Vascular plants	Melastomataceaegenus sp5
108	Vascular plants	Melastomataceaegenus sp6
109	Vascular plants	Melastomataceaegenus sp7
110	Vascular plants	Melastomataceaegenus sp8
111	Vascular plants	Melastomataceaegenus sp9
112	Vascular plants	Menispermaceaegenus sp1
113	Vascular plants	Menispermaceaegenus sp2
114	Vascular plants	Moraceaegenus sp1
115	Vascular plants	Orchidaceaegenus sp01
116	Vascular plants	Orchidaceaegenus sp02
117	Vascular plants	Orchidaceaegenus sp03
118	Vascular plants	Orchidaceaegenus sp04
119	Vascular plants	Orchidaceaegenus sp05
120	Vascular plants	Orchidaceaegenus sp06
121	Vascular plants	Orchidaceaegenus sp07
122	Vascular plants	Orchidaceaegenus sp08

123	Vascular plants	Orchidaceaegenus sp09
124	Vascular plants	Orchidaceaegenus sp10
125	Vascular plants	Poaceaegenus sp1
126	Vascular plants	Rubiaceaegenus sp1
127	Vascular plants	Rubiaceaegenus sp2
128	Vascular plants	Rubiaceaegenus sp3
129	Vascular plants	Sapindaceaegenus sp1
130	Vascular plants	Scrophulariaceaegenus sp1
131	Vascular plants	Gardenescapezebrastripe sp4
132	Vascular plants	Lycianthescf sp1
133	Vascular plants	Lycianthescf sp2
134	Vascular plants	Solanaceaegenus sp05
135	Vascular plants	Solanaceaegenus sp06
136	Vascular plants	Solanaceaegenus sp07
137	Vascular plants	Solanaceaegenus sp08
138	Vascular plants	Solanaceaegenus sp09
139	Vascular plants	Solanaceaegenus sp10
140	Vascular plants	Aegiphilacf sp1
141	Vascular plants	Vitaceaegenus sp1
142	Vascular plants	Helminotheca prolifera
143	Vascular plants	Cf casalpinaceae
144	Vascular plants	Cf lauraceae
145	Vascular plants	Cf moraceae
146	Vascular plants	Indetgen1 indetsp1
147	Vascular plants	Indetgen4 indetsp4

148	Vascular plants	Indetgen6 indetsp6
149	Vascular plants	Indetgen2 indetsp2
150	Vascular plants	Indetgen3 indetsp3
151	Vascular plants	Tree a
152	Vascular plants	Tree uk
153	Vascular plants	Unknown yellow
154	Vascular plants	Yellow composite

Supplementary Table 4. Percentages of local assemblages with non-native species across all taxa and for each taxon separately.

Taxa	Number of assemblages with non-natives	Total number of assemblages	Percentage (%)
Ants	113	407	27.8
Birds	457	4925	9.3
Mammals	293	1147	25.5
Spiders	182	773	23.5
Vascular plants	1406	4461	31.5
Across all taxa	2451	11713	20.9

Supplementary Table 5. Odds ratios of non-native species incidence in local assemblages in response to the interaction of LU-type and LU-intensity. LU-types were Primary (primary vegetation), Secondary (secondary vegetation), Plantation, Pasture, Cropland and Urban (urban areas). LU-intensity was categorized as minimal use, light use and intense use. Odds ratios are back-transformed pairwise contrasts to the reference level 'Primary vegetation under Minimal use' (=1). Asterisks indicate significant differences (p values: *<0.05; **<0.01 and ***<0.001) compared to the reference level. Significant values are highlighted in bold. The NA indicates that intense use in urban areas was removed from the analysis since all of the assemblages have non-native species.

**LU-type**	**LU-intensity**	**Assemblage(n)**	**Odds.ratio**	**SE**	**p.value**
Primary	Minimal use	2759	1	-	-
	Light use	1282	1.4	0.2	0.95 ns
	**Intense use**	**325**	**3.8**	**1.3**	**0.02 ***
Secondary	**Minimal use**	**1391**	**4.4**	**0.9**	**6.4 e-11 *****
	Light use	656	2.3	0.6	0.08 ns
	**Intense use**	**243**	**5.5**	**2**	**0.0007 *****
Plantation	**Minimal use**	**331**	**6**	**2.4**	**0.001 *****
	**Light use**	**643**	**11.6**	**3.1**	**1.9 e-13 *****
	**Intense use**	**181**	**6.4**	**2.5**	**0.0002 *****
Pasture	**Minimal use**	**245**	**13.9**	**4.5**	**3.04 e-13 *****
	**Light use**	**391**	**6.9**	**1.8**	**7.7 e-12 *****
	**Intense use**	**67**	**8.3**	**4.3**	**0.005 ****
Cropland	**Minimal use**	**292**	**9.3**	**3.7**	**3.0 e-6 *****
	Light use	373	2.6	0.9	0.49 ns
	**Intense use**	**468**	**31.7**	**11**	**1.5 e-13 *****
Urban	**Minimal use**	**102**	**9.1**	**4.2**	**0.0002 *****
	**Light use**	**100**	**12.6**	**6.4**	**7.3 e-5 *****
	Intense use	NA	NA	NA	NA

Supplementary Table 6. Odds ratios of non-native species incidence in local assemblages in response to LU-type. LU-types were Primary (primary vegetation), Secondary (secondary vegetation), Plantation, Pasture, Cropland and Urban (urban areas). Primary was considered as reference level (=1). Asterisks indicate significant differences (p values: *<0.05; **<0.01 and ***<0.001; respectively) compared to the reference level (Primary vegetation). Significant values are highlighted in bold. 'NA's indicate that the respective levels could not be considered in the analysis due to low sample size.

	**LU**	**Assemblage (n)**	**Odds ratios**	**SE**	**p.value**
Across all taxa	Primary	4544	1	-	-
	**Secondary**	**2722**	**2.6**	**0.4**	**5.8 e-10*****
	**Plantation**	**1238**	**6**	**1.1**	**6.5 e-14*****
	**Pasture**	**1394**	**4.9**	**0.9**	**7.1 e-14*****
	**Cropland**	**1592**	**8.1**	**1.6**	**6.1 e-14*****
	**Urban**	**223**	**9**	**3.2**	**5.1 e-9*****
Ants	Primary	68	1	-	-
	Secondary	165	2.9	1.7	0.48 ns
	**Plantation**	**55**	**34.2**	**27.1**	**0.0001*****
	**Pasture**	**53**	**8.2**	**5.8**	**0.04 ***
	**Cropland**	**38**	**15.8**	**13.7**	**0.02 ***
	Urban	28	6.9	5.4	0.13 ns
Birds	Primary	1656	1	-	-
	Secondary	1003	2.2	0.8	0.20 ns
	**Plantation**	**704**	**4.3**	**1.3**	**1.8 e-5*****
	Pasture	615	2.9	1.3	0.21 ns
	**Cropland**	**776**	**11.5**	**3.6**	**1.9 e-13*****
	**Urban**	**171**	**15.8**	**8.2**	**1.6 e-6*****
Mammals	Primary	657	1	-	-
	**Secondary**	**263**	**6.8**	**2.6**	**3.7 e-6 *****
	**Plantation**	**148**	**3.4**	**1.5**	**0.02 ***
	Pasture	46	0.5	0.4	0.75 ns
	Cropland	NA	NA	NA	NA
	Urban	NA	NA	NA	NA
Spiders	Primary	95	1	-	-
	Secondary	192	1.7	1	0.81 ns
	Plantation	NA	NA	NA	NA
	**Pasture**	**85**	**157.3**	**128.9**	**4.1 e-9 *****
	Cropland	390	0.3	0.5	0.90 ns
	Urban	NA	NA	NA	NA

	Primary	2068	1	-	-
	**Secondary**	**1099**	**2**	**0.4**	**0.005 ****
Vascular plants	**Plantation**	**324**	**12.2**	**4**	**4.6 e-13*****
	**Pasture**	**595**	**2.9**	**0.8**	**0.002****
	**Cropland**	**367**	**3.1**	**0.9**	**0.0007*****
	Urban	NA	NA	NA	NA

Supplementary Table 7. Comparison of models of non-native incidence, number and proportion that do or do not include the assignment of assemblage locations to one of the 14 biomes distinguished in Supplementary References[13] into the random effects structure. The full model had the interaction of LU-type and LU-intensity as fixed factors (the assemblages classified as LU-intensity "Cannot decide" in PREDICTS were not included) while the LU-type model had LU-type as the only fixed factor. Random effects of the compared models were either study blocks nested in study sites (SSB/SS), or study blocks nested in study sites nested in biomes (SSB/SS/biome) (or in the cases where model selection had favoured a simpler random-effects structure SS vs. SS/biome). The statistical model test used are two-sided. The comparison was based on the difference in Akaike Information Criterion (AIC) values as well as on a likelihood-ratio test (LRT) comparing the two models. In the model of non-native species proportion, the biome 'Tropical & Subtropical Dry Broadleaf Forests' in birds was not included since it caused convergence problems due to low sample size (n=2).

Variables	Models	AIC values (best models)	AIC values (including biome)	AIC dif	p values (LRT)
Non-native incidence	Across all taxa (full model)	4961.599	4959.692	-1.907	0.04808
	Across all taxa (LU-type only)	5505.733	5505.212	-0.521	0.1123 (ns)
	Ants	337.4273	339.4273	2	0.9998 (ns)
	Birds	1451.384	1453.324	1.94	0.8067(ns)
	Mammals	623.7195	625.7194	2	0.9911(ns)
	Spiders	279.7961	281.796	1.999	0.9976(ns)
	Vascular plants	2492.729	2491.042	-1.687	0.05483 (ns)
Non-native species number	Across all taxa (full model)	7240.2	7242.156	1.956	0.9999(ns)
	Across all taxa (LU-type only)	7752.859	7754.859	2	1(ns)
	Ants	310.7193	312.7193	2	0.9999(ns)
	Birds	901.7968	903.7968	2	1(ns)
	Mammals	-	-	-	-
	Spiders	350.797	352.797	2	1(ns)
	Vascular plants	5060.121	5062.121	2	1(ns)

	Across all taxa (full model)	-4743.516	-4741.516	2	1(ns)
	Across all taxa (LU-type only)	-5013.137	-5012.872	0.265	0.1878(ns)
Non-native species proportions	Ants	-109.8102	-107.8102	2	1(ns)
	Birds	-1222.374	-1220.374	2	0.9999(ns)
	Mammals	-1565.954	-1563.954	2	1(ns)
	Spiders	-253.2297	-251.2297	2	1(ns)
	Vascular plants	-3488.011	-3486.053	1.958	0.8378(ns)

Supplementary Table 8. Cross-validation of robustness of coefficient estimates in the models of non-native incidence, number and proportion computed by excluding assemblages from one biome in turn. The values of coefficient estimates are back-transformed pairwise contrasts to the reference level 'primary vegetation under Minimal use' in the full model and to the reference level 'primary vegetation' in the subset model. Shown are mean estimates, mean standard errors of coefficient estimates and the 95% confidence intervals of mean coefficient estimates (lower CI and upper CI) across all the cross-validation runs (= number of biomes represented by assemblages). 'NA's indicate that the respective factor levels could not be included in the analysis due to low assemblage number. For non-native species number, mammals were not analyzed due to low variance in species number of assemblages. Spiders were not analyzed due to low sample size (see the details in Methods).

1) Odds ratios of non-native incidence

Full model for non-native occurrence across all taxa: fixed=LU-type *LU-intensity

LU-types	LU-intensity	Mean odds ratio	SE.ratio	Lower.CI	Upper.CI	Validation times (n)
Primary	Minimal use	1.0000	-	-	-	14
	Light use	1.3716	0.0593	1.2435	1.4997	14
	Intense use	3.8060	0.1672	3.4447	4.1673	14
Secondary	Minimal use	4.5014	0.1952	4.0797	4.9232	14
	Light use	2.3314	0.1071	2.1001	2.5628	14
	Intense use	5.3878	0.2356	4.8787	5.8968	14
Plantation	Minimal use	6.0262	0.1929	5.6095	6.4429	14
	Light use	13.2798	1.2792	10.5163	16.0434	14
	Intense use	6.6189	0.1100	6.3813	6.8566	14
Pasture	Minimal use	13.8202	0.6698	12.3731	15.2672	14
	Light use	6.8343	0.3952	5.9805	7.6882	14
	Intense use	8.5596	0.6138	7.2335	9.8857	14
Cropland	Minimal use	9.3297	0.6233	7.9832	10.6762	14
	Light use	3.1767	0.7439	1.5696	4.7838	14
	Intense use	32.6842	2.9255	26.3641	39.0043	14

	Minimal use	9.1295	0.4894	8.0723	10.1867	14
Urban	Light use	18.8221	6.1241	5.5919	32.0524	14
	Intense use	NA	NA	NA	NA	NA
Subset model across all taxa: fixed=LU-type						
Primary	-	1.0000	-	-	-	14
Secondary	-	2.6050	0.0870	2.4171	2.7929	14
Plantation	-	6.1446	0.2489	5.6069	6.6822	14
Pature	-	4.8472	0.2093	4.3951	5.2993	14
Cropland	-	8.3649	0.6024	7.0634	9.6664	14
Urban	-	9.2272	0.2518	8.6832	9.7711	14
Subset model for ants: fixed=LU-type						
Primary	-	1.0000	-	-	-	10
Secondary	-	3.1248	0.2729	2.5074	3.7422	10
Plantation	-	38.9691	6.6551	23.9142	54.0240	10
Pasture	-	8.6932	0.7939	6.8972	10.4891	10
Cropland	-	17.2236	1.3669	14.1314	20.3158	10
Urban	-	7.4461	0.6812	5.9052	8.9870	10
Subset model for birds: fixed=LU-type						
Primary	-	1.0000	-	-	-	12
Secondary	-	2.3122	0.1143	2.0606	2.5639	12
Plantation	-	4.1068	0.2475	3.5620	4.6516	12
Pasture	-	2.7199	0.1194	2.4572	2.9826	12
Cropland	-	10.9222	0.6606	9.4682	12.3763	12
Urban	-	15.2411	0.4975	14.1461	16.3361	12
Subset model for mammals: fixed=LU-type						
Primary	-	1.0000	-	-	-	9

Secondary	-	8.0382	1.7791	3.9357	12.1408	9
Plantation	-	3.2160	0.3706	2.3614	4.0706	9
Pasture	-	0.4076	0.0537	0.2838	0.5314	9
Cropland	-	NA	NA	NA	NA	NA
Urban	-	NA	NA	NA	NA	NA
Subset model for spiders: fixed=LU-type						
Primary	-	1.0000	-	-	-	4
Secondary	-	1.7725	0.0570	1.5911	1.9539	4
Plantation	-	NA	NA	NA	NA	NA
Pasture	-	151.2500	3.4416	140.2972	162.2027	4
Cropland	-	0.2848	0.1599	-0.2242	0.7938	4
Urban	-	NA	NA	NA	NA	NA
Subset model for vascular plants: fixed=LU-type						
Primary	-	1.0000	-	-	-	11
Secondary	-	2.0720	0.1760	1.6798	2.4642	11
Plantation	-	13.0585	1.1198	10.5634	15.5536	11
Pasture	-	3.2171	0.3677	2.3979	4.0363	11
Cropland	-	3.5935	0.5638	2.3373	4.8498	11
Urban	-	NA	NA	NA	NA	NA

2) Non-native species number

Full model across all taxa: fixed=LU-type *LU-intensity

LU	LUI	Mean.num	SE.num	Lower.CI	Upper.CI	14
Primary	Minimal use	1.1419	0.0213	1.0945	1.1893	14
	Light use	1.6281	0.0094	1.6072	1.6491	14

	Intense use	2.0565	0.0540	1.9363	2.1767	14
Secondary	Minimal use	2.1435	0.0531	2.0253	2.2617	14
	Light use	1.9114	0.0246	1.8565	1.9662	14
	Intense use	1.8497	0.0317	1.7790	1.9203	14
Plantation	Minimal use	2.3757	0.0832	2.1903	2.5610	14
	Light use	2.0131	0.0615	1.8760	2.1502	14
	Intense use	1.3497	0.0227	1.2991	1.4003	14
Pasture	Minimal use	1.8019	0.0285	1.7385	1.8654	14
	Light use	2.0279	0.0320	1.9565	2.0993	14
	Intense use	1.9775	0.0174	1.9387	2.0162	14
Cropland	Minimal use	2.1732	0.0228	2.1224	2.2241	14
	Light use	1.3802	0.0337	1.3050	1.4553	14
	Intense use	1.5610	0.0468	1.4566	1.6654	14
Urban	Minimal use	2.3145	0.0868	2.1212	2.5078	14
	Light use	2.2714	0.0732	2.1083	2.4346	14
	Intense use	2.3837	0.1108	2.1368	2.6307	14
Subset model across all taxa: fixed=LU-type						
Primary	-	1.3316	0.0205	1.2860	1.3773	11
Secondary	-	2.1389	0.0438	2.0414	2.2364	11
Plantation	-	2.0024	0.0293	1.9372	2.0676	11
Pasture	-	1.8650	0.0247	1.8099	1.9201	11
Cropland	-	1.6756	0.0387	1.5895	1.7617	11
Urban	-	2.2760	0.0634	2.1348	2.4173	11
Subset model for ants: fixed=LU-type						
Primary	-	1.0345	0.0188	0.9885	1.0805	7
Secondary	-	1.0552	0.0082	1.0351	1.0752	7

Plantation	-	1.6783	0.1451	1.3233	2.0334	7
Pasture	-	1.3321	0.0546	1.1984	1.4658	7
Cropland	-	1.1806	0.0354	1.0941	1.2672	7
Urban	-	1.0448	0.0108	1.0183	1.0712	7
Subset model for birds: fixed=LU-type						
Primary	-	1.5402	0.0941	1.2790	1.8015	5
Secondary	-	1.6194	0.1695	1.1487	2.0901	5
Plantation	-	1.5497	0.1082	1.2493	1.8500	5
Pasture	-	1.5321	0.1492	1.1179	1.9463	5
Cropland	-	1.6364	0.1077	1.3373	1.9356	5
Urban	-	2.1360	0.2065	1.5628	2.7092	5
Subset model for vascular plants: fixed=LU-type						
Primary	-	1.6355	0.0333	1.5612	1.7098	11
Secondary	-	2.9803	0.0687	2.8271	3.1335	11
Plantation	-	3.0215	0.1021	2.7940	3.2491	11
Pasture	-	2.2324	0.0445	2.1332	2.3315	11
Cropland	-	2.2069	0.0595	2.0743	2.3395	11
Urban	-	3.9968	0.3926	3.1221	4.8715	11

3) Non-native proportions

Full model across all taxa: fixed=LU-type *LU-intensity						
LU	LUI	Mean.prop	SE.prop	Lower.CI	Upper.CI	11
Primary	Minimal use	0.3587	0.0063	0.3445	0.3728	11
	Light use	0.3878	0.0085	0.3689	0.4066	11
	Intense use	0.4379	0.0068	0.4228	0.4530	11
Secondary	Minimal use	0.3889	0.0079	0.3712	0.4066	11

	Light use	0.3914	0.0079	0.3738	0.4091	11
	Intense use	0.4372	0.0028	0.4310	0.4434	11
Plantation	Minimal use	0.4045	0.0069	0.3890	0.4199	11
	Light use	0.4364	0.0093	0.4157	0.4570	11
	Intense use	0.4654	0.0045	0.4553	0.4754	11
Pasture	Minimal use	0.3913	0.0086	0.3722	0.4105	11
	Light use	0.4106	0.0086	0.3915	0.4297	11
	Intense use	0.4094	0.0070	0.3937	0.4251	11
Cropland	Minimal use	0.4659	0.0044	0.4561	0.4757	11
	Light use	0.4071	0.0059	0.3940	0.4201	11
	Intense use	0.4163	0.0089	0.3964	0.4362	11
Urban	Minimal use	0.4418	0.0067	0.4267	0.4568	11
	Light use	0.4477	0.0055	0.4354	0.4600	11
	Intense use	0.4614	0.0153	0.4272	0.4956	11
Subset model for overall: fixed=LU-type						
Primary	-	0.4396	0.0057	0.3973	0.4819	11
Secondary	-	0.4101	0.0081	0.3505	0.4698	11
Plantation	-	0.4465	0.0063	0.3998	0.4931	11
Pasture	-	0.3761	0.0076	0.3197	0.4325	11
Cropland	-	0.4117	0.0074	0.3568	0.4666	11
Urban	-	0.4512	0.0057	0.4093	0.4931	11
Subset model for ants: fixed=LU-type						
Primary	-	0.3865	0.0236	0.3287	0.4442	7
Secondary	-	0.3871	0.0193	0.3398	0.4344	7
Plantation	-	0.6909	0.0052	0.6781	0.7037	7
Pasture	-	0.4096	0.0227	0.3540	0.4652	7

Cropland	-	0.4475	0.0238	0.3893	0.5058	7
Urban	-	0.5010	0.0137	0.4674	0.5346	7
Subset model for birds: fixed=LU-type						
Primary	-	0.1486	0.0087	0.1246	0.1727	5
Secondary	-	0.1429	0.0206	0.0856	0.2003	5
Plantation	-	0.1563	0.0147	0.1154	0.1972	5
Pasture	-	0.1318	0.0062	0.1145	0.1491	5
Cropland	-	0.1827	0.0174	0.1345	0.2309	5
Urban	-	0.2047	0.0203	0.1483	0.2611	5
Subset model for mammals: fixed=LU-type						
Primary	-	0.6417	0.0149	0.6053	0.6782	7
Secondary	-	0.6412	0.0145	0.6057	0.6767	7
Plantation	-	0.6838	0.0236	0.6261	0.7415	7
Pasture	-	0.6979	0.0142	0.6614	0.7344	7
Cropland	-	NA	NA	NA	NA	NA
Urban	-	NA	NA	NA	NA	NA
Subset model for vascular plants: fixed=LU-type						
Primary	-	0.1626	0.0052	0.1511	0.1740	11
Secondary	-	0.2148	0.0068	0.1997	0.2299	11
Plantation	-	0.2296	0.0153	0.1955	0.2638	11
Pasture	-	0.1960	0.0077	0.1788	0.2132	11
Cropland	-	0.2359	0.0063	0.2217	0.2500	11
Urban	-	0.3675	0.0239	0.3143	0.4206	11

Supplementary Table 9. Comparison of model results for non-native incidence, number and proportions without and with including the size of the sampling area of assemblages as additional fixed-effects predictor (fixed=LU-type*LU-intensity+size; random=SSB/SS). Shown are estimated coefficients and their p-values. LU-types were Primary (primary vegetation), Secondary (secondary vegetation), Plantation, Pasture, Cropland and Urban (urban areas). LU-intensity was categorized as minimal use, light use and intense use. The reference level is Primary vegetation under Minimal use. Asterisks indicate significant differences (p values: *<0.05; **<0.01 and ***<0.001), given in bold) compared to the reference level.

1) For odds ratios of non-native incidence

Fixed	Without sampling area		With sampling area	
	Estimate	p values	Estimate	p values
(Intercept)	**-5.41**	**< e-16*** **	**-5.52**	**2.2 e-9*** **
LUSecondary	**1.48**	**4.2 e-13*** **	**1.52**	**4.6 e-13*** **
LUPlantation	**1.8**	**8.5 e-6*** **	**2.03**	**2.0 e-6*** **
LUCropland	**2.23**	**2.0 e-8*** **	**2.12**	**9.8 e-7*** **
LUPasture	**2.63**	**5.6 e-16*** **	**2.70**	**4.1 e-16*** **
LUUrban	**2.21**	**1.7 e-06*** **	**2.25**	**1.1 e-6*** **
LUILight use	0.32	0.07 ns	**0.38**	**0.04***
LUIIntense use	**1.34**	**0.0001*** **	**1.48**	**3.5 e-5*** **
Sampling size	-	-	0.03	0.82ns
LUSecondary:LUILight use	**-0.98**	**0.001** **	**-0.99**	**0.001** **
LUPlantation:LUILight use	0.33	0.48ns	0.03	0.96ns
LUCropland:LUILight use	**-1.61**	**0.004** **	**-1.88**	**0.002** **
LUPasture:LUILight use	**-1.02**	**0.003** **	**-1.08**	**0.002** **
LUUrban:LUILight use	0.01	0.99ns	-0.03	0.97ns
LUSecondary:LUIIntense use	**-1.13**	**0.01***	**-1.00**	**0.03***
LUPlantation:LUIIntense use	**-1.28**	**0.04***	**-1.88**	**0.005** **
LUCropland:LUIIntense use	-0.12	0.84ns	-0.14	0.82ns
LUPasture:LUIIntense use	**-1.86**	**0.005** **	**-2.35**	**0.0009*** **

2) For non-native species number

(Intercept)	-0.06	0.48ns	**-0.03**	0.88ns
LUSV	**0.65**	**<2e-16*** **	**0.63**	**8.6 e-16*** **
LUPlantation	**0.76**	**1.6 e-5*** **	**0.88**	**8.4 e-7*** **
LUPasture	**0.47**	**0.0002*** **	**0.48**	**0.0002*** **
LUCropland	**0.65**	**4.9 e-7*** **	**0.67**	**4.3 e-6*** **
LUUrban	**0.7**	**4.9 e-7*** **	**0.72**	**1.5 e-7*** **
LUILight use	**0.36**	**1.2 e-5*** **	**0.38**	**3.9 e-6*** **
LUIIntense use	**0.6**	**8.9 e-5*** **	**0.60**	**5.5 e-5*** **
Sampling size	-	-	-0.02	0.66ns
LUSecondary:LUILight use	**-0.48**	**8.9 e-5*** **	**-0.46**	**0.0002*** **
LUPlantation:LUILight use	**-0.57**	**0.007** **	**-0.63**	**0.003** **
LUPasture:LUILight use	-0.24	0.08ns	-0.26	0.07ns
LUCropland:LUILight use	**-0.82**	**6.0 e-12*** **	**-0.91**	**1.4 e-7*** **
LUUrban:LUILight use	**-0.37**	**0.04***	**-0.40**	**0.03***
LUSecondary:LUIIntense use	**-0.75**	**0.0002*** **	**-0.73**	**0.0002*** **
LUPlantation:LUIIntense use	**-1.19**	**7.9 e-6*** **	**-1.27**	**2.2 e-6*** **
LUPasture:LUIIntense use	**-0.51**	**0.04***	-0.45	0.11ns
LUCropland:LUIIntense use	**-0.95**	**1.9 e-7*** **	**-0.99**	**2.5 e-6*** **
LUUrban:LUIIntense use	**-0.61**	**0.014***	**-0.60**	**0.014***
3) For non-native species proportions				
(Intercept)	-1.28	**4.4 e-13*** **	**-0.92**	**0.02***
LUSV	0.19	0.053ns	0.18511	0.06ns
LUPlantation	0.28	0.21ns	0.33568	0.15ns
LUPasture	0.19	0.17ns	0.18366	0.21ns
LUCropland	**0.82**	**8.6 e-8*** **	**0.65107**	**0.0005*** **
LUUrban	**0.58**	**0.0003*** **	**0.58121**	**0.0003*** **
LUILight use	0.17	0.07ns	0.1789	0.07ns
LUIIntense use	**0.56**	**0.005** **	**0.56639**	**0.004** **

Sampling size	-	-	-0.06355	0.42ns
LUSecondary:LUILight use	-0.16	0.07ns	-0.11283	0.45ns
LUPlantation:LUILight use	0.03	0.89ns	0.03406	0.9ns
LUPasture:LUILight use	-0.04	0.80ns	-0.03049	0.84ns
LUCropland:LUILight use	**-0.68**	**0.0002*** **	**-0.54505**	**0.02***
LUUrban:LUILight use	-0.12	0.53ns	-0.13306	0.5ns
LUSecondary:LUIIntense use	-0.2	0.38ns	-0.19323	0.4ns
LUPlantation:LUIIntense use	-0.05	0.88ns	-0.07714	0.82ns
LUPasture:LUIIntense use	-0.43	0.12ns	-0.35689	0.27ns
LUCropland:LUIIntense use	**-1.03**	**6.5 e-5*** **	**-0.8618**	**0.002****
LUUrban:LUIIntense use	-0.19	0.55ns	-0.20243	0.53ns

Supplementary Table 10. Comparison of models of non-native incidence, number and proportion that either account or do not account for the location of the assemblage on an island or on the mainland. The statistical model test used are two-sided. The random-effects structure was SSB/SS/island_continent (SSB= study block, SS = study site) or, in the cases where model selection had favoured a simpler random-effects structure, SS/island_continent. The comparison was based on the difference in Akaike Information Criterion (AIC) values as well as on a likelihood-ratio test (LRT) comparing the two models. Some of the models had convergence problems due to low numbers of assemblages on islands and could hence not be fitted.

Variables	Models	AIC values (best models)	AIC values (including island-continent)	AIC difference	p values (LRT)
Non-native incidence	Across all taxa (full model)	4961.60	4961.99	0.39	0.2(ns)
	Across all taxa (LU-type model)	5505.73	5504.54	-1.20	0.07(ns)
	Ants	337.43	339.43	2.00	1(ns)
	Birds	1057.50	1059.40	1.90	0.76(ns)
	Mammals	623.72	625.72	2.00	0.99 (ns)
	Spiders	279.80	281.80	2.00	1(ns)
	Vascular plants	2492.73	2494.73	2.00	0.99 (ns)
Non-native species number	Across all taxa (full model)	7240.16	7242.16	2.00	1(ns)
	Across all taxa (LU-type model)	7752.86	7754.86	2.00	1(ns)
	Ants	310.72	312.72	2.00	1(ns)
	Birds	901.80	903.80	2.00	1(ns)
	Mammals	-	-	-	-
	Spiders	350.80	352.80	2.00	1(ns)
	Vascular plants	5060.12	5062.12	2.00	1(ns)
Non-native species proportion	Across all taxa (full model)	-4743.52	-4741.52	2.00	1(ns)

Across all taxa (LU-type model)	-5013.14	-5011.10	2.04	1(ns)
Ants	-	-	-	-
Birds	-1229.27	-1227.27	2.00	1(ns)
Mammals	-1565.95	-1563.95	2.00	1(ns)
Spiders	-253.23	-251.23	2.00	0.95(ns)
Vascular plants	-	-	-	-

Supplementary Table 11. Test of robustness of coefficient estimates in the models of non-native incidence, number and proportion computed by excluding assemblages from islands. The values of coefficient estimates are back-transformed pairwise contrasts to the reference level 'primary vegetation under Minimal use' in the full model and to the reference level 'primary vegetation' in theLU-type only model. Shown are mean estimates, mean standard errors of coefficient estimates across the two models without assemblages from islands and with the assemblages from islands. 'NA's indicate that the respective factor levels could not be considered in the models due to low sample size, lack of response variation (non-native species number in case of mammals), or problems with (spiders for models without assemblages from islands (see the details in Methods)).

**1) Odds ratios of non-native incidence**			
Full model for non-native incidence across all taxa: fixed=LU-type *LU-intensity			
LU-types	LU-intensity	Mean odds ratio	SE.ratio
Primary	Minimal use	1	-
	Light use	1.29	0.089
	Intense use	4.21	0.373
Secondary	Minimal use	4.9	0.487
	Light use	1.99	0.3
	Intense use	5.78	0.318
Plantation	Minimal use	4.91	1.112
	Light use	23.61	12.008
	Intense use	6.58	0.134
Pasture	Minimal use	13.01	0.933
	Light use	5.32	1.564
	Intense use	10.44	2.098
Cropland	Minimal use	10.07	0.756
	Light use	2.41	0.145
	Intense use	18.68	13.023
Urban	Minimal use	8.57	0.526
	Light use	12.43	0.177
	Intense use	NA	NA
Subset model across all taxa: fixed=LU-type			
Primary	-	1	-
Secondary	-	2.61	0.05
Plantation	-	6.4	0.408

Pasture	-	3.8	1.058
Cropland	-	5.99	2.15
Urban	-	8.3	0.738
Subset model for ants: fixed=LU-type			
Primary	-	1	-
Secondary	-	3.51	0.644
Plantation	-	33.06	1.162
Pasture	-	8.85	0.831
Cropland	-	15.79	0.039
Urban	-	8.73	1.799
Subset model for birds: fixed=LU-type			
Primary	-	1	-
Secondary	-	1.24	0.954
Plantation	-	3.89	0.43
Pasture	-	2.3	0.561
Cropland	-	8.06	3.443
Urban	-	14.31	1.48
Subset model for mammals: fixed=LU-type			
Primary	-	1	-
Secondary	-	8.47	1.72
Plantation	-	3.39	0.01
Pasture	-	0.51	0.05
Cropland	-	NA	NA
Urban	-	NA	NA
Subset model for spiders: fixed=LU-type			
Primary	-	1	-
Secondary	-	0.9	0.815
Plantation	-	NA	NA
Pasture	-	79.01	78.272
Cropland	-	0.15	0.132
Urban	-	NA	NA
Subset model for vascular plants: fixed=LU-type			
Primary	-	1	-
Secondary	-	1.94	0.06
Plantation	-	12.92	0.726
Pasture	-	3.9	0.953
Cropland	-	2.62	0.528
Urban	-	NA	NA
**2) Non-native species number**			

Full model across all taxa: fixed=LU-type *LU-intensity			
LU	**LUI**	Mean.num	SE.num
Primary	Minimal use	1.11	0.025
	Light use	1.71	0.093
	Intense use	2.08	0.028
Secondary	Minimal use	2.27	0.101
	Light use	1.97	0.047
	Intense use	1.91	0.051
Plantation	Minimal use	2.09	0.335
	Light use	1.97	0.001
	Intense use	1.44	0.103
Pasture	Minimal use	1.81	0.002
	Light use	1.97	0.064
	Intense use	1.97	0.004
Cropland	Minimal use	2.24	0.069
	Light use	1.39	0.017
	Intense use	1.54	0.019
Urban	Minimal use	2.31	0.041
	Light use	2.25	0.001
	Intense use	2.81	0.56
Subset model across all taxa: fixed=LU-type			
Primary	-	1.33	0.001
Secondary	-	2.22	0.069
Plantation	-	1.95	0.042
Pasture	-	1.76	0.106
Cropland	-	1.66	0.004
Urban	-	2.24	0.004
Subset model for ants: fixed=LU-type			
Primary	-	1.02	0.023
Secondary	-	1.05	0.019
Plantation	-	1.35	0.296
Pasture	-	1.38	0.005
Cropland	-	1.19	0.015
Urban	-	1.03	0.035
Subset model for birds: fixed=LU-type			
Primary	-	1.44	0.105
Secondary	-	1.29	0.285

Plantation	-	1.6	0.041
Pasture	-	1.26	0.257
Cropland	-	1.41	0.21
Urban	-	1.8	0.255
Subset model for vascular plants: fixed=LU-type			
Primary	-	1.48	0.149
Secondary	-	2.65	0.356
Plantation	-	2.44	0.534
Pasture	-	1.94	0.288
Cropland	-	1.91	0.252
Urban	-	3.16	0.916
**3) Non-native proportions**			
Full model across all taxa: fixed=LU-type *LU-intensity			
LU	LUI	Mean.prop	SE.prop
Primary	Minimal use	0.39	0.027
	Light use	0.42	0.031
	Intense use	0.46	0.019
Secondary	Minimal use	0.41	0.021
	Light use	0.41	0.023
	Intense use	0.46	0.023
Plantation	Minimal use	0.4	0.003
	Light use	0.45	0.025
	Intense use	0.48	0.018
Pasture	Minimal use	0.42	0.028
	Light use	0.44	0.029
	Intense use	0.43	0.019
Cropland	Minimal use	0.48	0.01
	Light use	0.42	0.017
	Intense use	0.42	0.01
Urban	Minimal use	0.46	0.017
	Light use	0.47	0.018
	Intense use	0.4	0.072
Subset model for overall: fixed=LUtype			
Primary	-	0.39	0.026
Secondary	-	0.42	0.021
Plantation	-	0.45	0.022

Pasture	-	0.42	0.025
Cropland	-	0.44	0.014
Urban	-	0.45	0.015
Subset model for ants: fixed=LU-type			
Primary	-	0.39	0.009
Secondary	-	0.39	0.006
Plantation	-	0.69	0.01
Pasture	-	0.41	0.004
Cropland	-	0.46	0.003
Urban	-	0.49	0.004
Subset model for birds: fixed=LU-type			
Primary	-	0.13	0.023
Secondary	-	0.1	0.031
Plantation	-	0.12	0.03
Pasture	-	0.11	0.016
Cropland	-	0.15	0.034
Urban	-	0.17	0.029
Subset model for mammals: fixed=LU-type			
Primary	-	0.67	0.034
Secondary	-	0.66	0.03
Plantation	-	0.72	0.032
Pasture	-	0.72	0.028
Cropland	-	NA	NA
Urban	-	NA	NA
Subset model for spiders: fixed=LU-type			
Primary	-	0.16	0.006
Secondary	-	0.13	0.044
Plantation	-	0.19	0.01
Pasture	-	0.18	0.06
Cropland	-	0.1	0.02
Urban	-	NA	NA
Subset model for vascular plants: fixed=LU-type			
Primary	-	0.16	0.008
Secondary	-	0.22	0.012
Plantation	-	0.22	0.003
Pasture	-	0.22	0.029
Cropland	-	0.24	0.012
Urban	-	0.38	0.008

Supplementary Table 12. Number of non-native species in local assemblages in response to the interaction of LU-type and LU-intensity. LU-types were Primary (primary vegetation), Secondary (secondary vegetation), Plantation, Pasture, Cropland and Urban (urban areas). LU-intensity was categorized as minimal use, light use and intense use. Number of non-native species was back-transformed. A compact letter display (CLD) indicates the significance of differences ($p<0.05$) between all pairwise comparisons of LU-type and LU-intensity combinations.

LU-type	LU-intensity	Assemblage (n)	Species number	SE	Group
Primary	Minimal use	411	1.1	0.1	a
	Light use	196	1.6	0.2	bcd
	Intense use	63	2.1	0.3	bcd
Secondary	Minimal use	392	2.2	0.2	b
	Light use	143	1.9	0.2	bcd
	Intense use	56	1.9	0.3	abcd
Plantation	Minimal use	33	2.4	0.4	bcd
	Light use	168	2	0.2	bcd
	Intense use	61	1.3	0.2	abcd
Pasture	Minimal use	101	1.8	0.2	bcd
	Light use	142	2	0.2	bc
	Intense use	38	2	0.4	abcd
Cropland	Minimal use	47	2.2	0.2	bc
	Light use	176	1.4	0.1	ad
	Intense use	126	1.5	0.2	acd
Urban	Minimal use	73	2.3	0.3	bcd
	Light use	71	2.2	0.4	bcd
	Intense use	17	2.2	0.5	abcd

Supplementary Table 13. Number of non-native species in local assemblages in response to LU-type. LU-types were Primary (primary vegetation), Secondary (secondary vegetation), Plantation, Pasture, Cropland and Urban (urban areas). Number of non-native species was back-transformed. A compact letter display (CLD) indicates the significance of differences ($p<0.05$) between all pairwise comparisons of LU-type levels.

	LU-type	Assemblage (n)	Species number	SE	Group
Across all taxa	Primary	674	1.3	0.1	a
	Secondary	629	2.2	0.2	b
	Plantation	283	2	0.2	bc
	Pasture	300	1.9	0.2	bc
	Cropland	403	1.7	0.1	c
	Urban	162	2.2	0.3	bc
Ants	Primary	6	1	0.5	a
	Secondary	33	1.1	0.2	a
	Plantation	38	1.6	0.4	a
	Pasture	13	1.4	0.4	a
	Cropland	10	1.2	0.4	a
	Urban	13	1.1	0.4	a
Birds	Primary	102	1.5	0.2	a
	Secondary	39	1.4	0.2	ab
	Plantation	79	1.6	0.2	ab
	Pasture	16	1.3	0.2	a
	Cropland	78	1.6	0.2	ab
	Urban	143	2	0.3	b
Spiders	Primary	13	1	0.2	a
	Secondary	79	1.2	0.1	a
	Plantation	7	1	0.3	ab
	Pasture	78	1.8	0.2	b
	Cropland	5	1	0.3	ab
Vascular plants	Primary	393	1.6	0.2	a
	Secondary	389	3	0.4	b
	Plantation	120	3	0.5	bc
	Pasture	189	2.2	0.3	bc
	Cropland	309	2.2	0.3	c
	Urban	6	4.1	1	bc

Supplementary Table 14. Proportion of non-native species in local assemblages in response to the interaction of LU-type and LU-intensity. LU-types were Primary (primary vegetation), Secondary (secondary vegetation), Plantation, Pasture, Cropland and Urban (urban areas). LU-intensity was categorized as minimal use, light use and intense use. Proportion of non-native species was back-transformed. A compact letter display (CLD) indicates the significance of differences ($p<0.05$) between all pairwise comparisons of LU-type and LU-intensity combinations

LU-type	LU-intensity	Assemblage (n)	Non-native proportion	SE	Group
Primary	Minimal use	411	0.34	0.03	a
	Light use	196	0.37	0.03	ab
	Intense use	63	0.43	0.03	abc
Secondary	Minimal use	392	0.37	0.03	ab
	Light use	143	0.37	0.03	ab
	Intense use	56	0.42	0.03	abc
Plantation	Minimal use	33	0.39	0.04	abc
	Light use	168	0.42	0.03	bc
	Intense use	61	0.45	0.02	bc
Pasture	Minimal use	101	0.37	0.03	abc
	Light use	142	0.39	0.03	abc
	Intense use	38	0.39	0.04	abc
Cropland	Minimal use	47	0.46	0.02	c
	Light use	176	0.39	0.03	abc
	Intense use	126	0.4	0.03	abc
Urban	Minimal use	73	0.43	0.03	bc
	Light use	71	0.43	0.03	abc
	Intense use	17	0.47	0.03	abc

Supplementary Table 15. Proportion of non-native species among all species in local assemblages in response to LU-type. LU-types were Primary (primary vegetation), Secondary (secondary vegetation), Plantation, Pasture, Cropland and Urban (urban areas). Proportion of non-native species was back-transformed. A compact letter display (CLD) indicates the significance of differences (p<0.05) between all pairwise comparisons of LU-type levels.

	LU-type	Assemblage (n)	Non-native proportion	SE	group
Across all taxa	Primary	674	0.38	0.03	a
	Secondary	629	0.41	0.02	b
	Plantation	283	0.44	0.02	bc
	Pasture	300	0.41	0.03	abc
	Cropland	403	0.44	0.02	c
	Urban	162	0.45	0.02	bc
Ants	Primary	6	0.34	0.1	a
	Secondary	33	0.34	0.08	a
	Plantation	38	0.72	0.08	a
	Pasture	13	0.37	0.09	a
	Cropland	10	0.44	0.1	a
	Urban	13	0.48	0.1	a
Birds	Primary	102	0.16	0.03	abc
	Secondary	39	0.13	0.02	a
	Plantation	79	0.15	0.03	ab
	Pasture	16	0.13	0.03	abc
	Cropland	78	0.18	0.03	c
	Urban	143	0.2	0.03	bc
Mammals	Primary	160	0.63	0.06	a
	Secondary	89	0.63	0.06	a
	Plantation	39	0.69	0.07	a
	Pasture	4	0.69	0.11	a
Spiders	Primary	13	0.17	0.04	a
	Secondary	79	0.17	0.03	a
	Plantation	7	0.2	0.07	a
	Pasture	78	0.24	0.04	a
	Cropland	5	0.12	0.05	a
Vascular plants	Primary	393	0.16	0.02	a
	Secondary	389	0.21	0.03	b
	Plantation	120	0.22	0.03	b

Pasture	189	0.19	0.03	ab
Cropland	309	0.23	0.03	b
Urban	6	0.38	0.05	c

Supplementary Table 16. Number of all assemblages and of those with at least one non-native species, given separately for the LU-types. LU-types were Primary (primary vegetation), Secondary (secondary vegetation), Plantation, Pasture, Cropland and Urban (urban areas).

	LU-type	Number of assemblages with non-native species	Total number of assemblages
Ants	Primary	6	68
	Secondary	33	165
	Plantation	38	55
	Pasture	13	53
	Cropland	10	38
	Urban	13	28
Birds	Primary	102	1656
	Secondary	39	1003
	Plantation	79	704
	Pasture	16	615
	Cropland	78	776
	Urban	143	171
Mammals	Primary	160	657
	Secondary	89	263
	Plantation	39	148
	Pasture	4	46
	Cropland	1	21
	Urban	NA	12
Spiders	Primary	13	95
	Secondary	79	192
	Plantation	7	7
	Pasture	78	85

	Cropland	5	390
	Urban	NA	4
Vascular plants	Primary	393	2068
	Secondary	389	1099
	Plantation	120	324
	Pasture	189	595
	Cropland	309	367
	Urban	6	8
Across all taxa	Primary	674	4544
	Secondary	629	2722
	Plantation	283	1238
	Pasture	300	1394
	Cropland	403	1592
	Urban	162	223

Supplementary References

1. Hudson, L. N. et al. The PREDICTS database: A global database of how local terrestrial biodiversity responds to human impacts. Ecol Evol 4, 4701–4735 (2014).
2. Newbold, T. et al. Global effects of land use on local terrestrial biodiversity. Nature 520, 45–50 (2015).
3. Guénard, B., Weiser, M. D., Gomez, K., Narula, N., & Economo, E. P. The Global Ant Biodiversity Informatics (GABI) database: synthesizing data on the geographic distribution of ant species (Hymenoptera: Formicidae). Myrmecological News 24, 83-89 (2017).
4. Dyer, E. E., Redding, D. W., & Blackburn, T. M. The global avian invasions atlas, a database of alien bird distributions worldwide. Scientific Data, 4, 1-12 (2017).
5. Biancolini, D., Vascellari, V., Melone, B., Blackburn, T. M., Cassey, P., Scrivens, S. L., & Rondinini, C. DAMA: the global Distribution of Alien Mammals database, Ecology, e03474 (2021).
6. Blackburn, T., Scrivens, S., Heinrich, S., & Cassey, P. Patterns of selectivity in introductions of mammal species worldwide. NeoBiota, 33,33-51 (2017).
7. GRIIS: https://griis.org/

8. ICUN: https://www.iucnredlist.org
9. Sales, L.P., Ribeiro, B.R., Hayward, M.W., Paglia, A., Passamani, M. and Loyola, R. Niche conservatism and the invasive potential of the wild boar. *Journal of Animal Ecology*, *86*, 1214-1223 (2017).
10. Dawson, W. *et al.* Global hotspots and correlates of alien species richness across taxonomic groups. *Nat Ecol Evol* **1**, 1–7 (2017).
11. van Kleunen, M. *et al.* Global exchange and accumulation of non-native plants. *Nature* **525**, 100–103 (2015).
12. Pysek, P., J. Pergl, F. Essl, B. Lenzner, W. Dawson, H. Kreft, P. Weigelt et al. "Naturalized alien flora of the world: species diversity, taxonomic and phylogenetic patterns, geographic distribution and global hotspots of plant invasion. Preslia89: 203-274." (2017).
13. Olson, D.M., Dinerstein, E., Wikramanayake, E.D., Burgess, N.D., Powell, G.V., Underwood, E.C., D'amico, J.A., Itoua, I., Strand, H.E., Morrison, J.C. and Loucks, C.J., Terrestrial Ecoregions of the World: A New Map of Life on EarthA new global map of terrestrial ecoregions provides an innovative tool for conserving biodiversity. BioScience, 51, 933-938 (2001).
