# Supplementary material for: The impact of land use on non-native species incidence and number in local assemblages worldwide
Source: Nat Commun. 2023 Apr 12;14:2090. doi: 10.1038/s41467-023-37571-0 (PMC10097616; doi:10.1038/s41467-023-37571-0)
Supplement: Supplementary file 3 — Reporting Summary [file 41467_2023_37571_MOESM3_ESM.pdf]

nature portfolio

Corresponding author(s): Daijun Liu

Last updated by author(s): Mar 14, 2023

# Reporting Summary

Nature Portfolio wishes to improve the reproducibility of the work that we publish. This form provides structure for consistency and transparency in reporting. For further information on Nature Portfolio policies, see our Editorial Policies and the Editorial Policy Checklist.

## Statistics

For all statistical analyses, confirm that the following items are present in the figure legend, table legend, main text, or Methods section.

| n/a | Confirmed | |
|---|---|---|
| ☐ | ☒ | The exact sample size ($n$) for each experimental group/condition, given as a discrete number and unit of measurement |
| ☐ | ☒ | A statement on whether measurements were taken from distinct samples or whether the same sample was measured repeatedly |
| ☐ | ☒ | The statistical test(s) used AND whether they are one- or two-sided<br>*Only common tests should be described solely by name; describe more complex techniques in the Methods section.* |
| ☐ | ☒ | A description of all covariates tested |
| ☐ | ☒ | A description of any assumptions or corrections, such as tests of normality and adjustment for multiple comparisons |
| ☐ | ☒ | A full description of the statistical parameters including central tendency (e.g. means) or other basic estimates (e.g. regression coefficient) AND variation (e.g. standard deviation) or associated estimates of uncertainty (e.g. confidence intervals) |
| ☐ | ☒ | For null hypothesis testing, the test statistic (e.g. $F$, $t$, $r$) with confidence intervals, effect sizes, degrees of freedom and $P$ value noted<br>*Give P values as exact values whenever suitable.* |
| ☐ | ☒ | For Bayesian analysis, information on the choice of priors and Markov chain Monte Carlo settings |
| ☐ | ☒ | For hierarchical and complex designs, identification of the appropriate level for tests and full reporting of outcomes |
| ☐ | ☒ | Estimates of effect sizes (e.g. Cohen's $d$, Pearson's $r$), indicating how they were calculated |

*Our web collection on statistics for biologists contains articles on many of the points above.*

## Software and code

Policy information about availability of computer code

| | |
|---|---|
| Data collection | Data on local species assemblages (i.e. communities) were obtained from the published PREDICTS database (https://data.nhm.ac.uk/dataset/the-2016-release-of-the-predicts-database). Five taxonomic groups for which information on alien species occurrences at the regional scale was available in established databases: ants , birds, mammals, spiders and vascular plants. We also uploaded the data used to produce our results, i.e. the lists of species identified as alien in the PREDICTS assemblages (https://github.com/liudyuk/data-and-code-for-alien-invasion-in-local-assembalge.git). |
| Data analysis | The code for the analysis and making graph 1-4 are in the open access of Github: https://github.com/liudyuk/data-and-code-foralien-invasion-in-local-assembalge.git. The emmeans test is analyzed with the version 1.7.2.<br>All the analyses are conducted in R version 4.1.1. All details are described in the R code and main text. |

For manuscripts utilizing custom algorithms or software that are central to the research but not yet described in published literature, software must be made available to editors and reviewers. We strongly encourage code deposition in a community repository (e.g. GitHub). See the Nature Portfolio guidelines for submitting code & software for further information.

## Data

Policy information about availability of data

All manuscripts must include a data availability statement. This statement should provide the following information, where applicable:

- Accession codes, unique identifiers, or web links for publicly available datasets
- A description of any restrictions on data availability
- For clinical datasets or third party data, please ensure that the statement adheres to our policy

Data on local species assemblages (i.e. communities) were obtained from the published PREDICTS database (https://data.nhm.ac.uk/dataset/the-2016-release-of-the-predicts-database). The raw data for five taxonomic groups are protected and are not available due to data privacy laws. We uploaded the data for ants, birds, mammals, spiders and vascular plants. used to produce our results, i.e. the lists of species identified as alien in the PREDICTS assemblages.
github (https://github.com/liudyuk/data-and-code-for-alien-invasion-in-local-assembalge.git).

## Human research participants

Policy information about studies involving human research participants and Sex and Gender in Research.

| | |
|---|---|
| Reporting on sex and gender | Not used in this study |
| Population characteristics | Not used in this study |
| Recruitment | Not used in this study |
| Ethics oversight | Not used in this study |

Note that full information on the approval of the study protocol must also be provided in the manuscript.

# Field-specific reporting

Please select the one below that is the best fit for your research. If you are not sure, read the appropriate sections before making your selection.

☐ Life sciences ☐ Behavioural & social sciences ☒ Ecological, evolutionary & environmental sciences

For a reference copy of the document with all sections, see nature.com/documents/nr-reporting-summary-flat.pdf

# Ecological, evolutionary & environmental sciences study design

All studies must disclose on these points even when the disclosure is negative.

| | |
|---|---|
| Study description | Here, we address this issue by linking a global dataset of local assemblages collected in six LU-types used with three LU-intensity levels (the PREDICTS database) with data on the regional distribution of alien species from five taxonomic groups — ants, birds, mammals, spiders and vascular plants. |
| Research sample | PREDICTS is a compilation of data from 666 original studies published in 480 different sources (sampling period 1984-2013) and contains species lists of 26,114 local assemblages.<br>We considered five taxonomic groups for which information on non-native species naturalized at the regional scale was available in established databases: ants55, birds47, mammals33, spiders56, and vascular plants17,57. The latest versions of these databases document 303 ant, 361 bird, 239 mammal, 207 spider, and 15,111 vascular plant species naturalized as non-native in regions stated in the databases, respectively. |
| Sampling strategy | Prior to model selection, we tested for over- or under-dispersion with the DHARMa package version 0.4.5. The test results did not show any significance, indicating none of them have the over- or under-dispersion. |
| Data collection | We used the existed databases of PREDICTS which are compiled from 480 studies and non-native species distribution data for ants, birds, mammals, spiders and vascular plants which are provided by the co-authors. We identified the species as non-native to a particular region in each assemblage by matching species lists of local assemblages with the lists of species names. All analyses were conducted in R version 4.1.1 (R Core Team 2021). |
| Timing and spatial scale | The sampling period of PREDICTS is from 1984-2013 and at a global scale. Alien species distribution data are collected for the occurrence after 1500. |
| Data exclusions | The species were not identified that have been described in the Supplementary Table 3. |
| Reproducibility | All the data for the analyses and code for making figures1-4 are available for readers. |


March 2021

| | |
|---|---|
| Randomization | The randomization was not relevant to the work. |
| Blinding | Blinding was not applicable in this submission process. |

Did the study involve field work? ☐ Yes ☒ No

# Reporting for specific materials, systems and methods

We require information from authors about some types of materials, experimental systems and methods used in many studies. Here, indicate whether each material, system or method listed is relevant to your study. If you are not sure if a list item applies to your research, read the appropriate section before selecting a response.

## Materials & experimental systems

| n/a | Involved in the study |
|---|---|
| ☒ | ☐ Antibodies |
| ☒ | ☐ Eukaryotic cell lines |
| ☒ | ☐ Palaeontology and archaeology |
| ☒ | ☐ Animals and other organisms |
| ☒ | ☐ Clinical data |
| ☒ | ☐ Dual use research of concern |

## Methods

| n/a | Involved in the study |
|---|---|
| ☒ | ☐ ChIP-seq |
| ☒ | ☐ Flow cytometry |
| ☒ | ☐ MRI-based neuroimaging |
